# Transgenic Soybean for Production of Thermostable α-Amylase

**DOI:** 10.3390/plants13111539

**Published:** 2024-06-02

**Authors:** Zhenyan Cao, Ye Jiang, Jiajie Li, Ting Zheng, Chaoyang Lin, Zhicheng Shen

**Affiliations:** 1 State Key Laboratory of Rice Biology, Institute of Insect Sciences, College of Agriculture and Biotechnology, Zhejiang University, Hangzhou 310058, China; s13065707970@163.com (Z.C.); 17280115509@163.com (Y.J.); 22216268@zju.edu.cn (J.L.); lehaax@zju.edu.cn (T.Z.); chylin@zju.edu.cn (C.L.); 2 Zhongyuan Institute, Zhejiang University, Zhengzhou 450000, China

**Keywords:** α-amylase, *Bacillus stearothermophilus*, bioreactor, soybean, molecular farming, transgenic plant

## Abstract

Alpha-amylases are crucial hydrolase enzymes which have been widely used in food, feed, fermentation, and pharmaceutical industries. Methods for low-cost production of α-amylases are highly desirable. Soybean seed, functioning as a bioreactor, offers an excellent platform for the mass production of recombinant proteins for its ability to synthesize substantial quantities of proteins. In this study, we generated and characterized transgenic soybeans expressing the α-amylase AmyS from *Bacillus stearothermophilus*. The α-amylase expression cassettes were constructed for seed specific expression by utilizing the promoters of three different soybean storage peptides and transformed into soybean via *Agrobacterium*-mediated transformation. The event with the highest amylase activity reached 601 U/mg of seed flour (one unit is defined as the amount of enzyme that generates 1 micromole reducing ends per min from starch at 65 °C in pH 5.5 sodium acetate buffer). The optimum pH, optimum temperature, and the enzymatic kinetics of the soybean expressed enzyme are similar to that of the *E. coli* expressed enzyme. However, the soybean expressed α-amylase is glycosylated, exhibiting enhanced thermostability and storage stability. Soybean AmyS retains over 80% activity after 100 min at 75 °C, and the transgenic seeds exhibit no significant activity loss after one year of storage at room temperature. The accumulated AmyS in the transgenic seeds represents approximately 15% of the total seed protein, or about 4% of the dry seed weight. The specific activity of the transgenic soybean seed flour is comparable to many commercial α-amylase enzyme products in current markets, suggesting that the soybean flour may be directly used for various applications without the need for extraction and purification.

## 1. Introduction

Alpha-amylase (E.C.3.2.1.1) catalyzes the hydrolysis of internal 1,4-glycosidic linkages in starch, forming low molecular weight polymerization units such as glucose, maltose, and maltotriose. This class of amylases is widely used in textile, detergent, beverage, paper, pharma, bakery, and biofuel industries, constituting nearly 25–30% of the global market share of total enzymes [1,2]. α-Amylase demand is expected to contribute to its maximum share in the near future. The global industrial enzymes market, valued at USD 7.4 billion in 2023, is projected to reach USD 10.2 billion by 2028 (https://www.marketsandmarkets.com/PressReleases/industrial-enzymes.asp) (accessed on 30 May 2024). Although traditional microbial expression systems have been improved continuously, they can still fall short of cost and capacity requirements [3].

In several industrial processes, such as biofuel production, α-amylases must operate under harsh conditions, including elevated temperatures. Therefore, improving the thermostability of α-amylases is crucial for enhancing their industrial applicability and efficiency [4]. Various strategies, including protein engineering [5], immobilization techniques [6], and exploring extremophilic microorganisms [7], have been employed to enhance the thermostability of α-amylases. Enhancing the thermostability of α-amylases presents a multifaceted challenge and is highly desired in industrial applications.

Transgenic plants as bioreactors show immense potential for producing large quantities of proteins at a lower cost compared to traditional methods like microbe fermentation [8,9,10]. Although recombinant proteins can be functionally expressed in various plants, the soybean seed-based platform is highlighted by its natural ability to accumulate extremely large amounts of proteins (~40% protein content) and its suitability for long-term storage of functionally recombinant proteins [11,12,13]. Plant bioreactors offer several advantages, including reduced costs and easy scalability through well-established cultivation, harvesting, processing, and manufacturing protocols. Furthermore, the plant-based system eliminates the risk of human pathogens, microbial toxins, prions, or oncogenic sequences [14,15,16]. Recombinant amylases have been expressed in narbon bean, a legume species, but the amylase yield was insufficient for economical mass production [17]. Thus, exploring soybean as a bioreactor for large-scale α-amylase production is worthwhile, given its efficient and economic production system.

Seed-specific promoters with robust transcriptional activity have been utilized for achieving high expressing levels and restricting the localization of recombinant protein to seeds. Soybean seed storage proteins are categorized into four distinct groups based on their sedimentation coefficients: 2S, 7S, 11S, and 15S globulins [18]. Among these, β-conglycinin (7S) and glycinin (11S) account for 70% of seed storage proteins. In the endoplasmic reticulum, the β-conglycinin protein is synthesized, folded, and polymerized into a 156 kDa trimer, consisting of α, α′, and β subunits [19,20]. On the other hand, the 2S globulin represents approximately 8% of the total protein in soybean seeds and is encoded by a single gene. Obviously, the promoters of these storage proteins are suitable candidates for driving the expression of recombinant proteins in soybean seeds.

Here, we report the creation of transgenic soybean with a transgene of a thermostable α-amylase, AmyS, derived from *Bacillus stearothermophilus*, under the control of the promoters of 2S globulin, as well as the α and α’ subunit of the 7S β-conglycinin. We demonstrate that significant amounts of the α-amylase accumulated in the transgenic soybean seeds, which can be utilized in various applications without any extraction and purification.

## 2. Results

### 2.1. Generation of Transgenic Soybean Plants

**Figure 1:**
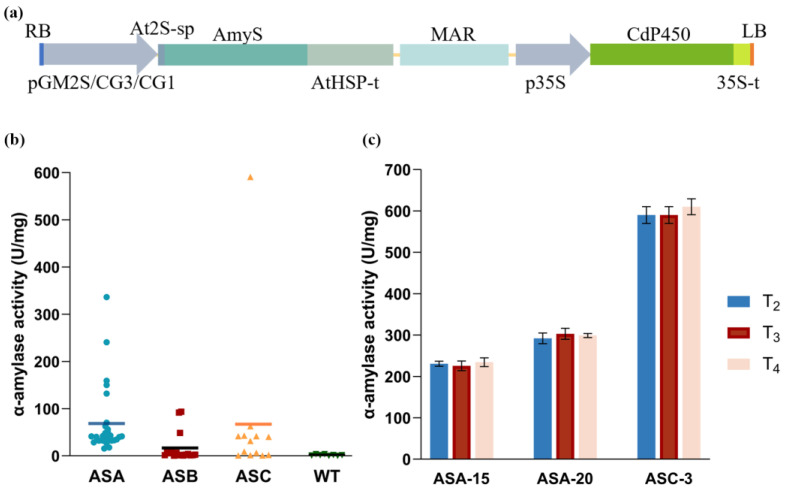
(**a**) Diagram of T-DNA used for soybean transformation. RB, T-DNA right border; pGM2S, soybean seed 2S globulin promoter; pCG3, α subunit of β-conglycinin promoter; pCG1, α′ subunit of β-conglycinin promoter; At2S-sp, *Arabidopsis* At2S signal peptide; AmyS, *Bacillus stearothermophilus* α-amylase gene; AtHSP-t, *Arabidopsis* heat shock protein 18.2 terminator; MAR, *Nicotiana tabacum* Rb7 matrix attachment region sequence; p35S, *Cauliflower mosaic* virus 35S promoter; CdP450, flazasulfuron resistance gene as a selection marker; 35S-t, *Cauliflower mosaic* virus 35S terminator; LB, T-DNA left border. (**b**) α-amylase activity of transgenic events with different promoters. ASA, events with soybean seed 2S globulin promoter; ASB, events with α subunit of β-conglycinin promoter; ASC, events with α′ subunit of β-conglycinin promoter; WT, wild type soybean seeds; (**c**) α-amylase activity of three generations (T_2_–T_4_) of the three highest expression events.

To create transgenic soybean expressing AmyS in seeds, multiple seed-specific promoters were evaluated for their effectiveness in driving seed specific expression. The promoters of the 2S glycinin (GM2S), the α subunit (CG3), and the α′ subunit (CG1) of the 7S β-conglycinin were obtained by PCR from genomic DNA of soybean cultivar “Tianlong 1”. Specifically, the GM2S promoter is 988 bp in length, CG1 promoter is 1278 bp, and CG3 promoter is 923 bp. The coding sequence of AmyS (without its original secretion signal peptide) was optimized for soybean expression and subsequently synthesized. The AmyS expression cassette is composed of a seed-specific promoter, the coding sequence for the *Arabidopsis* 2S albumin (At2S) signal peptide and AmyS, and the *Arabidopsis* heat shock protein 18.2 (AtHSP) terminator. The At2S signal peptide is anticipated to direct the synthesized protein to the endoplasmic reticulum [21,22,23]. The *Nicotiana tabacum* Rb7 matrix attachment region (MAR) was added to the 3’ end of the AmyS expression cassette. MAR has been shown to enhance gene expression and reduce position-effect variations in the transgenic host [24]. The T-DNAs also included a herbicide resistance gene (CdP450) expression cassette used for the selection of transgenic events in tissue culture and field cultivation [25]. A total of three T-DNA constructs with distinct seed-specific promoters were constructed (Figure 1a), designated as ASA (with GM2S promoter), ASB (with CG3 promoter), and ASC (with CG1 promoter), respectively.

Transgenic soybean plants were generated by *Agrobacterium*-mediated transformation with soybean cultivar “Tianlong 1”as the recipient. Multiple independent transgenic events were generated for each vector. DNS (3,5-dinitrosalicylic acid)-based colorimetric assay was performed to determine the α-amylase activity for the T_0_ seeds of transgenic soybeans, with wild-type (WT) soybean serving as the negative control. As expected, no significant α-amylase activity was detected in WT soybean seeds. The average enzyme activity for ASA, ASB, and ASC events were 68.6, 16.9, and 53.4 U/mg, respectively (one unit was defined as the amount of enzyme that generates 1 micromole reducing ends per min from starch at 65 °C, in pH 5.5 sodium acetate buffer) (Figure 1b). Among all the events, ASA-15, ASA-20, and ASC-3 displayed the highest activity levels, with α-amylase activity of 241.0, 336.5, and 591.1 U/mg of dry seed flour, respectively.

### 2.2. Characterization of the T-DNA Insertion of ASC-3

**Figure 2:**
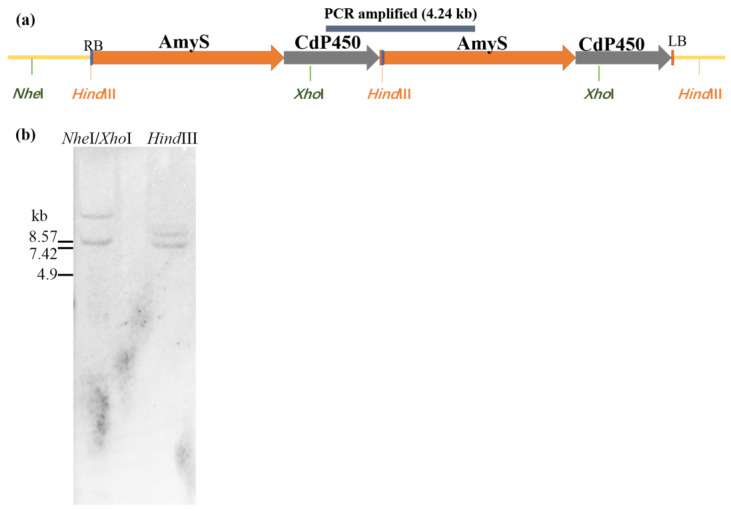
Characterization of T-DNA insert of event ASC-3. (**a**) Diagram of the two copies of T-DNA linked by head to tail. The fragment about 4.24 kb around the joint of the two T-DNA copy was amplified by PCR and confirmed by sequencing. (**b**) Southern blot analysis of ASC-3. DNA extracted from ASC-3 was probed by the DIG-labelled coding region of the *AmyS* gene. The estimated sizes of the signals on the Southern blot matched the expected sizes of 12.5 and 8.2 kb for *Nhe*I/*Xho*I digestion, and 9.3 and 8.2 kb for *Hind*III digestion.

The event ASC-3 was identified as the highest expresser among all the events generated and selected for further characterization. To determine the T-DNA copy number in the genome, Southern analysis was performed. The genomic DNA was isolated from the homozygous T_4_ plants and digested with *Hind*III or with double enzyme *Nhe*I and *Xho*I. The probe was made from the α-amylase gene AmyS. Two distinct bands were observed in both restriction digestions of the genomic DNA (Figure 2b), suggesting that two *AmyS* genes were integrated in the ASC-3 genome. Considering the 3:1 segregation ratio observed in the T_1_ plants (Table S1), we postulated that the two T-DNAs in the ASC-3 may be directly linked in a single insertion.

To investigate the T-DNA insertion of ASC-3, the flanking sequences of the inserted T-DNA were identified using high-efficiency thermal asymmetric interlaced PCR (hi-TAIL-PCR) [26]. Both the right border and left border flanking genomic sequence were obtained and confirmed by PCR test using one primer at T-DNA and another primer at genomic. Interestingly, the hi-TAIL-PCR also generated a product containing the sequences of the left border joined with right border, suggesting that the T-DNA insert may contain two copies of T-DNA linked by head to tail (Figure 2a). To further confirm the structure of the inserted two copies of T-DNA, a PCR was designed to amplify a larger DNA fragment that is across the possible joint of two T-DNAs. A 4.2 kb fragment, encompassing a 1.24 kb CdP450 expression cassette at the left side of the T-DNA and a 3.0 kb AmyS expression cassette at the right side of the T-DNA, was successfully amplified and confirmed by sequencing. The existence of this PCR product indicated that the two copies of T-DNAs were linked by head to tail at the insertion. Southern blot analysis showed that size of the bands agrees with the prediction based on the flanking genomic sequence and the insert, as illustrated in Figure 2a. *Hind*III makes a single cut within each T-DNA copy and is predicted to generate two DNA fragments of 9.3 and 8.2 kb. Similarly, *Nhe*I/*Xho*I makes a single cut within each T-DNA copy and is predicted to generate two DNA fragments of 12.5 and 8.3 kb. In conclusion, ASC-3 is a single insertion event with two copies of T-DNA linked by head to tail.

### 2.3. Characterization of Recombinant AmyS

**Figure 3:**
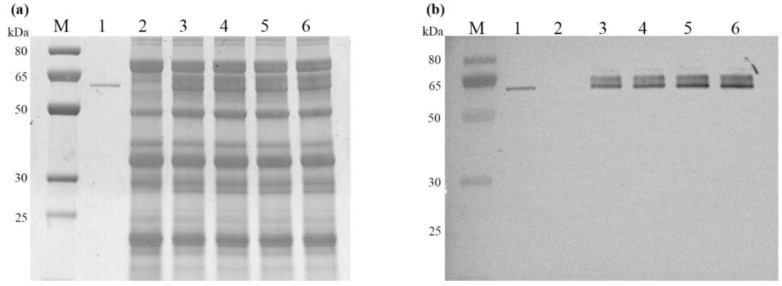
Analysis of AmyS in ASC-3 soybean seeds. Total proteins from four batches of ASC-3 T_3_ generation seeds (lane 3–6) were analyzed by SDS-PAGE (**a**) and by Western blot analysis with antibodies against AmyS (**b**). *E. coli* expressed AmyS served as a positive control (lane 1) while the sample from wild-type soybean served as a negative control (lane 2). Molecular size markers are shown in kDa on the left.

To investigate AmyS expression in seeds, total proteins extracted from four batches of ASC-3 seeds were analyzed by SDS-PAGE. Protein bands, approximately 60 kDa in size, which are close to the expected size of recombinant AmyS, were observed in transgenic soybean seeds but not in non-transgenic WT seeds (Figure 3a). To confirm that these transgenic seed-specific proteins were AmyS, Western blot was performed using antibodies against AmyS. As expected, a similar pattern of bands was detected at approximately the same size in the transgenic samples but not in the non-transgenic samples, suggesting these bands are AmyS proteins (Figure 3b). It was unexpected that two major bands, instead of one sharp band, were observed. We postulated that this was due to the difference in glycosylation. Grayscale scanning of the SDS-PAGE bands estimated that the abundance of AmyS protein in ASC-3 was approximately 15% of the total seed protein (Figure 3a).

**Figure 4:**
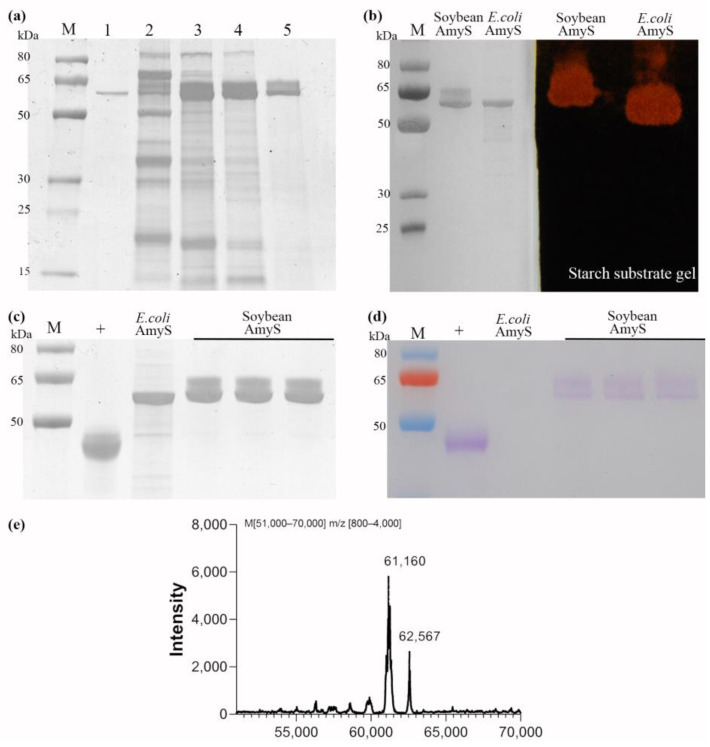
Characterization of AmyS protein from soybean seed. (**a**) SDS-PAGE analysis of AmyS samples from different purification steps. Lane 1, E coli expressed AmyS; Lane 2, total protein of transgenic seeds; Lane 3, proteins precipitated with (NH_4_)_2_SO_4_ at 30–40% of saturation; Lane 4, active sample eluted from Phenyl-Sepharose 6 Fast Flow HIC column; Lane 5, purified AmyS after passing through Q-Bestarose FF IEC column. M, molecular weight markers. (**b**) Starch substrate gel detection of soybean AmyS activity. The *E. coli* expressed and soybean expressed AmyS were separated by SDS-PAGE. Half of the gel was stained by Coomassie blue (left), and the other half was electrically transferred into a gel containing starch. After renaturation by soaking in TritonX 100, the starch gel was stained with KI/I_2_ solution (right). The visible reddish areas indicate the starch was digested by AmyS. (**c**,**d**) Glycosylation detection. *E. coli* expressed and soybean expressed AmyS were electrophoresed and stained with Coomassie blue (**c**) or with the Glycoprotein Staining Kit (**d**). Peroxidase from horseradish was used as positive control (+). (**e**) Molecular weight analysis of the soybean expressed AmyS proteins by LC/MS.

To further characterize the process, recombinant AmyS was purified from transgenic seeds using a three-step process. The soluble crude extract from the transgenic seeds was first precipitated with a 30–40% saturation of (NH_4_)_2_SO_4_. The pellet which contained the majority of AmyS activity was dissolved and separated by hydrophobic interaction chromatography on a Phenyl-Sepharose 6 Fast Flow column. The eluted fraction with AmyS activity was then further dialyzed to remove salt and subsequently passed through an anion exchange Q-Bestarose FF column. SDS-PAGE analysis of the samples from each step suggested that this purification procedure was effective (Figure 4a). Noticeably, the purified protein does not appear to be a single sharp band but a broad strap consisting of at least two major bands, which agrees with observations from the SDS-PAGE analysis of the crude extracts and the Western blot analysis (Figure 4a).

To confirm that the purified protein was recombinant AmyS, an SDS-PAGE substrate gel activity assay was conducted. The purified protein was first separated by SDS-PAGE and transferred onto a polyacrylamide gel containing 1% starch using Western blotting style transfer. After renaturing of the proteins by soaking in 1% Trion X-100, the substrate gel was stained with KI/I_2_ solution. Clearly visible reddish areas appeared at the same position where the purified AmyS proteins were present (Figure 4b), suggesting that the purified protein did have amylase activity.

Further, N-terminal sequences of the two major bands were determined by Edman degradation. We found that the N-terminal sequences of the sister bands are the same and identical to the expected sequence, confirming both bands were the same amylase protein. Furthermore, the total purified protein was analyzed by Nano LC-ESI-MS/MS and it was found that the amino acid sequences of the purified protein match well with the theoretical sequence of AmyS (Figure S1).

We also performed Liquid Chromatography/Mass Spectrometry (LC/MS) analysis to measure the molecular weight of the purified AmyS. The purified soybean AmyS was found to contain two major proteins with molecular weights of 61.16 and 62.57 kDa, respectively (Figure 4e). These molecular weights are significantly higher than the theoretical weight of 58 kDa, suggesting possible protein modification by glycosylation. To further confirm the glycosylation, a Glycoprotein Staining Kit (Thermo Fisher Scientific, Waltham, MA, USA), a modified method of the Periodic Acid-Schiff, was utilized to detect the glycosylation. As demonstrated in Figure 4c,d, the soybean recombinant AmyS was distinctly stained by the Schiff’s reagent, unlike the *E. coli* expressed AmyS. We concluded that soybean AmyS is glycosylated. Moreover, glycosylation appears to be uneven, as different molecular weights of the soybean recombinant AmyS were observed. NetOGlyc and NetNGlyc online tools were also employed to predict potential N-glycosylation and O-glycosylation sites within the amino acid sequence of AmyS. Seven potential N-glycosylation sites and six potential O-glycosylation sites were identified. However, further investigation is required to fully understand the specific types and quantities of glycans in the glycosylated AmyS proteins.

### 2.4. Enzymatic Properties of Recombinant AmyS

The enzymatic characteristics of the recombinant AmyS from soybean ASC-3 seed and *E. coli* were determined. The Lineweaver–Burk plot showed that the substrate affinity of AmyS remains unchanged when expressed in soybean seed. The Km values, as calculated from the Lineweaver–Burk plot, were 28.71 mg/mL for *E. coli* AmyS and 25.88 mg/mL for the purified soybean AmyS. The Vmax values for starch hydrolysis were 4.26 and 3.70 mL/min for the amylase expressed from *E. coli* and soybean, respectively (Table 1; Figure S2). The specific activity of purified soybean recombinant AmyS was 15,057 U/mg, comparable to the purified *E. coli* expressed AmyS, 17,300 U/mg (Table 1).

**Figure 5:**
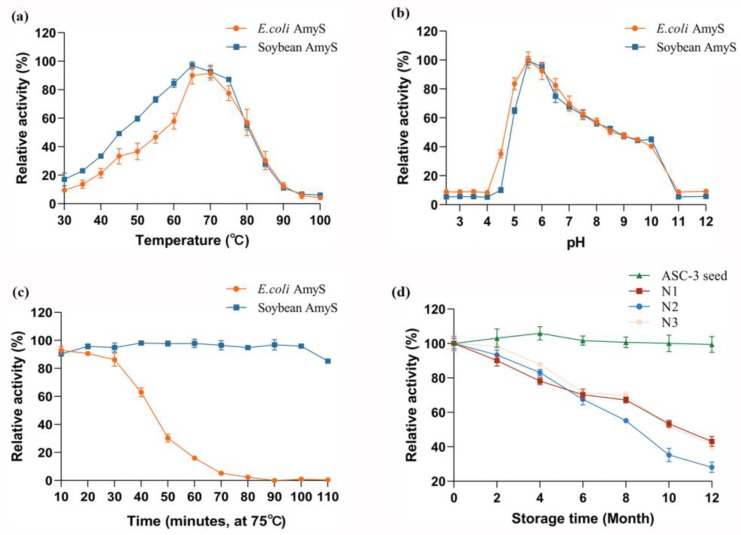
Enzymatic properties of α-amylase AmyS produced by soybean. Effect of temperature (**a**), pH (**b**), and time of incubation at high temperature (**c**) on activity of AmyS expressed from *E. coli* and soybean. (**d**) Comparison of the soybean expressed enzyme with three commercial thermostable enzymes (N1–N3) from different vendors at different storage times at room temperature.

The optimum temperature of the soybean recombinant AmyS α-amylase is 65 °C, which is the same as that of *E. coli* expressed enzyme (Figure 5a). The optimum pH is 5.5, consistent with the *E. coli* expressed AmyS (Figure 5b). Interestingly, the soybean AmyS exhibits significantly higher thermal stability than *E. coli* AmyS. The soybean expressed AmyS retained more than 80% of the enzymatic activities after 100 min of incubation at 75 °C, while the *E. coli* AmyS shows a dramatic loss of activity after just 30 min (Figure 5c). The storage stability of soybean AmyS in seeds was also evaluated. Several thermostable commercial enzymes with similar optimum temperatures produced by microbe fermentation from different vendors (N1–N3) were used for comparison. These commercial enzyme products incurred significant levels of activity loss after storage at room temperature. Only approximately 30–40% of enzyme activity was retained after storage for twelve months (Figure 5d). In contrast, the ASC-3 seeds exhibited no significant loss of enzyme activity under similar conditions. This demonstrates that soybean seeds are optimal for storage of the enzyme over a long period of time.

### 2.5. Accumulation and Tissue-Specific Expression of AmyS in Transgenic Soybean

**Figure 6:**
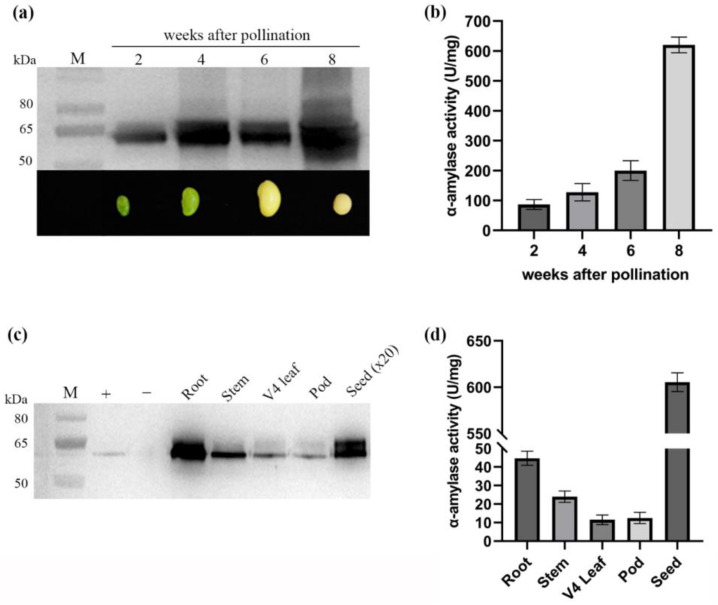
Western blot detection and enzyme activity measurement of AmyS in seeds at different time points after pollination (2, 4, 6, and 8 weeks) and in different tissues (root, stem, V4 leaf, pod, and seed). (**a**,**c**) Western detection, (**b**,**d**) activity measurement.

The accumulation of AmyS protein in soybean seed was assessed through Western blot analysis and measurement of α-amylase activity. The Western blot results revealed continuous accumulation of AmyS protein throughout the development of the soybean seed with a significant surge observed at 8 weeks after pollination (Figure 6a). The α-amylase activity of ASC-3 seeds at 2, 4, 6, and 8 weeks after pollination corroborated well with the Western blot analysis results. The average α-amylase activities of ASC-3 seeds sharply increased from 200 to 620 U/mg from 6 weeks to 8 weeks after pollination (Figure 6b).

To examine if AmyS is expressed tissue-specifically in soybean, different tissues from T_3_ plants of the ASC-3 event were analyzed. Western blot analysis revealed that AmyS was predominately expressed in seeds. Only very low levels of AmyS were detected in the roots, stems, V4 leaves, and pods of the transgenic plants (Figure 6c). Despite AmyS expression in all evaluated tissues, a 13-fold higher enzymatic activity was observed in seeds (Figure 6d).

### 2.6. Agronomic Traits and Yield Performance of Transgenic Plants

Previous reports have indicated that plant-based bioreactors may significantly influence plant agronomic traits when producing high levels of recombinant proteins. Additionally, research on plants expressing starch hydrolases has suggested a potential disruption of normal plant metabolism and resulted in altered phenotypes [27,28,29]. This disruption may be attributed to the recombinant enzymes, which have substantial activity at room temperature and can potentially impact starch and sugar metabolism.

The agronomic traits of the ASC-3 event, including germinating rate, plant height, number of nodes and branches, pods of per plant, total number of seeds, average seed weight, and days to maturity, were investigated. The ASC-3 event and its recipient soybean Tianlong 1 were planted in the same plot under identical conditions in a field in Hangzhou, China. Our findings revealed that almost all parameters, including emergence rate, plant height, number of nodes and branches, pods per plant, total seeds, weight of single or hundred seeds, and days to maturity, were similar between the ASC-3 event and Tianlong 1 (Table 2). The only exception was the number of nodes, which was slightly lower (13%) in the transgenic plants. In conclusion, the genetically engineered soybean ASC-3 showed minimal negative impact on its agronomic traits.

## 3. Discussion

### 3.1. Soybean Is a Suitable Bioreactor for Amylase Mass Production

The demand of α-amylase is substantial and continues to grow due to its crucial role in starch hydrolysis for various industrial processes, ranging from food, fermentation, detergent, pharmaceutical, brewing, textile, and paper industries. Currently, α-amylase is manufactured by microbial fermentation, which necessitates equipment, culture medium and energy sources such as electricity. New low-cost production methods are highly desired. Transgenic crop bioreactors for the production of large quantities of amylase could offer a more economical solution. Previously, amylases have been successfully expressed in transgenic plants such as maize, rice, alfalfa, narbon bean, tobacco, and potato [29,30,31,32,33]. In tobacco leaves, the expression levels reach up to 0.3% TSP [31]. Recombinant α-amylase in transgenic potato leaves was estimated to be 0.06% TSP, a protein level adequate only to liquefy starch in its transgenic storage-roots [29]. It is likely that seeds could produce more concentrated enzymes than other plant tissues like leaves. However, the study on expression of α-amylase in narbon bean seeds did not achieve a high enough activity level to be utilized as an enzyme source to substitute for fermentation enzymes. The amount of enzyme expressed in the narbon bean seeds was only capable of degrading its endogenous starch in the seeds [17]. Even in the case of transgenic corn Enogen, which expresses a hyper-thermostable α-amylase Amy797E and is the only transgenic product that has been successfully commercialized, its seeds only contain 1.0 to 1.3 mg amylase per gram of dry seeds, or 0.1~1.3% of the seed weight [32,34]. Significantly higher expression is required to significantly reduce production cost. In this study, the ASC-3 transgenic soybean seeds contain approximately 4% α-amylase AmyS of the dry seed weight, which is much higher than any other attempts on other plants. Soybean appears to have a greater potential to express large amounts of amylase in its seeds. This is not surprising, as soybean is capable of expressing extremely large quantities of proteins, up to 40% of dry seed weight. Furthermore, soybean seeds contain very low amounts of starch, and therefore the amylase expression and accumulation are less likely to disrupt the normal seed physiology. In contrast, strong amylase activity from a transgene will likely liquify the storage starch automatically during the seed growing stages in starch rich seeds such as corn, rice, and wheat. When we tried to express the AmyS in corn and rice, we found that the starch in the seeds was liquefied, which resulted in a big yield loss and difficulty to harvest (Figure S3 and [33]). In the case of Enogen, its recombinant amylase Amy797E has very little activity at 40 °C or lower temperatures; therefore, its starch in seeds is not digested by the amylase. Overall, soybean seeds, which typically contains less than 1% starch, are likely one of the most suitable crops as a bioreactor for amylase production.

### 3.2. ASC-3 Is a Cost-Effective Amylase Production Line

In this study, we demonstrated that soybean is an excellent bioreactor for producing recombinant α-amylase. The transgenic soybean ASC-3 seeds exhibited an α-amylase activity of 601 U/mg dry seed weight, and the recombinant α-amylase proteins accounted for 15% total seed protein, or about 4% of the dry weight in seeds. The high expression of ASC-3 may be the result of the tandem repeat of two T-DNAs. The α-amylase specific activity of ASC-3 soybean flour is comparable to that of various commercial thermostable α-amylases currently in markets (Figure S4). This indicates that ASC-3 seed flour can be directly used as a product for industrial processes without the need for extraction and purification. The expenses related to downstream processing, including protein concentration and purification, can account for over 80% of the total cost in enzyme production [3,35]. The amylase production method reported here significantly simplifies the production process, avoiding microbial cultures as well as protein purification processes for most applications. Therefore, the transgenic soybean described in this report is likely to be a more cost-effective alternative to microbial fermentation.

### 3.3. Glycosylated Amylase May Be More Thermostable

Interestingly, the enzyme expressed in soybean showed significant improved thermostability and storage stability compared to the same enzyme expressed by *E. coli*, while its enzymatic kinetics and other properties such as optimum pH and temperature remained similar. This improvement is likely due to the glycosylation of the soybean produced enzyme.

Both glycosylation staining analysis and molecular weight analysis strongly suggest that the α-amylase AmyS produced in soybean seeds is glycosylated. Glycosylation, a common post-translational modification in eukaryotes, was found to impact various enzyme functions [36,37]. Srivastava obtained a glycosylated *Bacillus* sp. α-amylase via the cyanogen bromide activation method and found that the glycosylated enzyme exhibited better thermal stability compared to the native enzyme, while the optimum temperature and optimum pH remained virtually unchanged [38]. This observation aligns with the findings of our study. The enhanced thermostability is likely ascribed to the decreased flexibility in the protein structure induced by glycosylation, which subsequently leads to an improvement in both the structural and thermal stability of the enzyme [39,40]. Additionally, we found no significant loss of activity of the α-amylase in the soybean seeds after one year of storage, while the commercial amylase produced by microbial fermentation lost about 60–70% of its activity (Figure 5d). Recombinant human proinsulin in transgenic seeds showed little or no degradation after storage at room temperature for 7 years [41]. The long storage life may result from both glycosylation and the favorable micro-environmental storage conditions within the seeds.

## 4. Materials and Methods

### 4.1. Cloning of Seed-Specific Promoters from Soybean

The seed-specific promoters of the 2S globulin GM2S (GLYMA_13G288100), the α subunit CG3 (GLYMA_20G148400), and α’ subunit CG1 (GLYMA_10G246300) were amplified from soybean cultivar “Tianlong 1” using PCR with the following primers: GM2SF (5’-CAGATTAAACGACGCCGTTTCG-3’)/GM2SR (5’-GAAGTGAAGGTGGGGGCATTTATTG-3’), CG1F (5’-GACTAATGCCTGATTAGTTGACATGACGA-3’)/CG1R (5’-AGTATATCTTAAATTCTTTAATACGGTG-3’) and CG3F (5’-CGAACTACGAGTTATGAAGTGTCAAT-3’)/CG3R (5’-AGTATCTTAAATTATTTATTAAGGT-3’). The PCR products were initially cloned into the pMD18-T vector (TaKaRa, Tokyo, Japan), and their sequences were verified by sequencing. The corresponding promoter vectors were named pGEM-GM2S, pGEM-CG1, and pGEM-CG3, respectively.

### 4.2. Construction of the T-DNA Vectors for Soybean Transformation

The amino acid sequence of the α-amylase AmyS was derived from *Bacillus stearothermophilus* (GenBank accession A24549). The coding sequence of the mature peptide of AmyS (without its original secretion signal peptide) was optimized for soybean expression and synthesized by Shanghai Sangon Limited Corp (Shanghai, China) with the following modification: an *Xba*I site and the sequence encoding the *Arabidopsis* 2S albumin signal peptide (GenBank accession NP_194445) were added at the 5’ end of the *AmyS* gene; an *Arabidopsis* heat shock protein 18.2 terminator (GenBank accession At5g59720), *Nicotiana tabacum* Rb7 matrix attachment region (GenBank accession U67919), and a *Kpn*I site were added to the 3’ end of the *AmyS* gene.

The binary T-DNA transformation plasmid was constructed based on pCAMBIA1300. First, an intermediate vector pCAMBIA1300-P450 was created by replacing the hygromycin-resistant gene with the flazasulfuron-resistant cytochrome *P450* gene from Bermuda grass between *Xho*I sites [25]. A three-way ligation was carried out with pCAMBIA1300-P450 predigested with *Hind*III and *Kpn*I as the vector backbone. One fragment was the seed-specific promoter predigested with *Hind*III and *Xba*I from the promoter vectors (pGEM-GM2S, pGEM-CG1, and pGEM-CG3). The other fragment was the AmyS sequence (including signal peptide, coding region, terminator and MAR) predigested with *Xba*I and *Kpn*I. The resulting T-DNA vectors with different promoters were named ASA, ASB, and ASC, respectively.

### 4.3. Soybean Transformation

The binary vectors ASA, ASB, and ASC were introduced into *Agrobacterium tumefaciens* strain EHA105 by electroporation. Soybean transformation was carried out by Agrobacterium-mediated transformation of soybean half-seed cotyledonary explants, following the protocol described by Paz [42]. Briefly, “Tianlong 1” soybean seeds were surface sterilized using chlorine for 16 h. After 12 h germination, the cotyledonary explants were isolated and inoculated with *Agrobacterium* for 3 h. The explants were then co-cultivated for 5 days. Shoot initiation and elongation medium were supplemented with 0.1 mg/L and 0.04 mg/L flazasulfuron to select for transformants, respectively. The rooted plantlets were transferred to soil, acclimated, and grown to maturity in a greenhouse at 26 °C, with 16 h of light and 8 h of darkness. T_0_ plants were screened by PCR analysis for the presence of *AmyS* gene.

### 4.4. Determination of α-Amylase Activity

The α-amylase activity was determined by measuring the enzymatic release of reducing sugars from starch [43]. One unit of enzyme activity (U) was defined as the amount of enzyme producing 1 μmol of reducing ends per minute, measured as maltose equivalents. Reducing ends released in the reaction were estimated by the 3′5-dinitrosalicylic (DNS) acid method using a maltose standard curve. Briefly, transgenic soybean tissues were homogenized in 20 mM sodium acetate buffer (pH 5.5) containing 250 mM NaCl, and the homogenate was used for the activity assay. The assay mixture, containing 50 µL homogenate and 450 µL 1% soluble starch (Solarbio, Beijing, China) in 20 mM sodium phosphate buffer (pH 5.5) was incubated at 65 °C for 1 h. After incubation, 1 mL of DNS solution was added to stop the reaction. The mixture was boiled for 5 min and the absorbance measured at 540 nm. Reaction controls (samples with substrate and enzyme react for 0 min) were run in parallel. Each reaction was replicated three times for each assay. The enzyme activity data reported is the average of three technical and two-to-four biological replications. The tissue-specific α-amylase activity was determined after subtracting the background activity from WT samples.

The α-amylase activity was determined by measuring the enzymatic release of reducing sugars from starch [43]. One unit of enzyme activity (U) was defined as the amount of enzyme producing 1 μmol of reducing ends per minute, measured as maltose equivalents. Reducing ends released in the reaction were estimated by the 3′5-dinitrosalicylic (DNS) acid method using a maltose standard curve. Briefly, transgenic soybean tissues were homogenized in 20 mM sodium acetate buffer (pH 5.5) containing 250 mM NaCl, and the homogenate was used for the activity assay. The assay mixture, containing 50 µL homogenate and 450 µL 1% soluble starch (Solarbio, Beijing, China) in 20 mM sodium phosphate buffer (pH 5.5) was incubated at 65 °C for 1 h. After incubation, 1 mL of DNS solution was added to stop the reaction. The mixture was boiled for 5 min and the absorbance measured at 540 nm. Reaction controls (samples with substrate and enzyme react for 0 min) were run in parallel. Each reaction was replicated three times for each assay. The enzyme activity data reported is the average of three technical and two-to-four biological replications. The tissue-specific α-amylase activity was determined after subtracting the background activity from WT samples.

### 4.5. Identification of T-DNA Flanking Sequences by hiTAIL-PCR

The left and right border flanking sequences of the T-DNA from the selected transformants were obtained by the. high-efficiency TAIL-PCR (hiTAIL-PCR) method [26]. The PCR primers and conditions were the same as reported [26]. The tertiary hiTAIL-PCR products were cloned into pEASY-T1 vector (TransGen Biotech, Beijing, China) and sequenced.

### 4.6. Southern Analysis

Genomic DNA from transgenic and WT soybean was obtained using a CTAB-based protocol. Approximately 25 µg genomic DNA was digested with *Hind*III or *Nhe*I/*Xho*I and then separated on a 0.8% agarose gel. The DNA was denatured, neutralized, and then transferred onto a Hybond-N^+^ membrane (Amersham-Pharmacia Biotech, Uppsala, Sweden) using the capillary transfer method. The AmyS specific probe was prepared from PCR amplified full-length DNA, encoding the AmyS gene using the DIG High Prime DNA Labeling and Detection Starter Kit II according to the instruction of the manufacturer (Roche, Germany). The primers used for AmyS amplification were ASF (5′-AGCTGCACCGTTCAACGGCACC-3′) and ASR (5′-GTGCTCACGGTGGTCTTCCTC-3′). Chemiluminescent signal detection was performed with the FluorChem FC2 Imaging System (Cell Biosciences, Santa Clara, CA, USA).

### 4.7. Expression and Purification of AmyS Protein from E. coli

The AmyS gene was constructed into the expression vector pET28a (Novagen, San Diego, CA, USA) with a His tag fused to its C-terminus. The expression vector was transformed into *E. coli* BL21 (DE3) strain (Stratagene, Santa Clara, CA, USA). A single *E. coli* colony harboring the pET28a-AmyS vector was picked and incubated in Luria–Bertani (LB) liquid medium at 37 °C with shaking at 220 rpm. When the OD_600_ of the culture reached 0.6, isopropyl β-D-1-thiogalactopyranoside (IPTG) was added into the LB medium to a 1 mM final concentration for inducing expression for 12 h at 16 °C with shaking at 220 rpm. The cells were harvested by centrifugation at 8000× *g* for 5 min at 4 °C, resuspended in 1X PBS, then lysed by sonication on ice. The supernatant of the lysates was collected after centrifugation at 12,000× *g* at 4 °C for 10 min. The *E. coli* expressed AmyS protein was purified from the supernatant using a Ni^2+^-NTA agarose column (Qiagen, Valenica, CA, USA) according to the manufacturer’s instructions.

### 4.8. Purification of the α-Amylase in Transgenic Soybean Seeds

Transgenic soybean seed flour (5 g/batch) was mixed with extraction buffer (20 mM sodium acetate, containing 250 mM NaCl, pH 5.5) at a 20:1 (*v*/*w*) ratio. Extraction was carried out in a 250 mL beaker with a magnetic stirrer at 100 rpm at room temperature for 3 h. The suspension was allowed to settle overnight or centrifuged at 12,000× *g* rpm for 10 min. Subsequently, the supernatant was collected, and the protein was precipitated using (NH_4_)_2_SO_4_ saturation (30–40%). The pellet, which contained the majority of the amylase activity, was resuspended in 50 mM phosphate buffer (pH 7.0) for the chromatography.

A Phenyl-Sepharose 6 Fast Flow column (Amersham Biosciences, Uppsala, Sweden), a hydrophobic interaction resin, was equilibrated with 25 mL binding buffer (600 mM (NH_4_)_2_SO_4_, 50 mM phosphate buffer, pH 7.0). After loading the sample, unbound proteins were washed out with the binding buffer. The bound proteins were eluted by stepwise decreasing the concentration of (NH_4_)_2_SO_4_ (480, 360, 240, 120, 0 mM) in 50 mM phosphate buffer, pH 7.0. Eluent from each (NH_4_)_2_SO_4_ concentration was collected in 10 mL fractions.

The eluted fractions with α-amylase activity were selected and combined. The active samples were then dialyzed against 50 mM phosphate buffer (pH 7.0) for 24 h with three buffer changes. An anion exchange chromatography column (Q-Bestarose FF, Bestchrom, Shanghai, China) was prepared and equilibrated with 25 mL 50 mM phosphate buffer (pH 7.0). The dialyzed sample was loaded directly onto the equilibrated column. The majority of the amylase activity was detected in the flow-through, which was found to be of high purity and was subsequently used for AmyS protein characterization and analysis.

### 4.9. Analysis of AmyS Proteins

#### 4.9.1. SDS-PAGE

Total soybean seed proteins were extracted from 10 mg of dry seed powder with 1 mL of 1X protein SDS-PAGE loading buffer (1% SDS, 10 mM Tris-HCl, pH 6.8, 50 mM DTT, 10% glycerol, 5% β-mercaptoethanol, 0.008% bromophenol blue), followed by boiling at 100 °C for 10 min. The sample was centrifuged for 5 min at 12,000× *g* rpm, and the supernatant was collected for analysis. Five µL of the sample was loaded onto 4–20% surePAGETM precast gel (GenScript, Piscataway, NJ, USA) and subjected to electrophoresis at 200 V for 30 min. Coomassie Brilliant Blue R-250 (GenScript, Piscataway, NJ, USA) was used to stain the protein bands.

#### 4.9.2. Western Blot

For Western blot analysis, proteins were treated and separated by SDS-PAGE as described above. Subsequently, the proteins were transferred onto polyvinylidene fluoride (PVDF) membranes using the eBlotTM L1 transfer system (GenScript, Piscataway, NJ, USA). The membranes were blocked in 5% skim milk in PBST for 1 h at room temperature. Rabbit serum against AmyS as the primary antibody was utilized (1:1000 dilution). Goat anti-rabbit IgG (H&L) (HRP) (Abcam, Cambridge, UK) served as the secondary antibody (1:8000 dilution). Finally, the protein bands were visualized using enhanced chemiluminescence (ECL) substrates.

#### 4.9.3. Characteristic of Soybean AmyS Protein

For Western blot analysis, the sample proteins were treated and separated by SDS-PAGE as described above. Subsequently, the proteins were transferred onto polyvinylidene fluoride (PVDF) membranes using the eBlotTM L1 transfer system (GenScript, Piscataway, NJ, USA). The membranes were blocked in 5% skim milk in PBST for 1 h at room temperature. Rabbit serum against AmyS as the primary antibody was utilized (1:1000 dilution). Goat anti-rabbit IgG (H&L) (HRP) (Abcam, Cambridge, UK) served as the secondary antibody (1:8000 dilution). Finally, the protein bands were visualized using enhanced chemiluminescence (ECL) substrates.

#### 4.9.4. Starch Substrate Gel

Purified soybean AmyS protein (10 µg) was mixed with protein SDS-PAGE loading buffer and boiled at 100 °C for 5 min. After protein separation by 4–20% SDS-PAGE, the gel was divided into two parts. One part, containing the samples and molecular markers, was stained with Coomassie brilliant blue R-250. The same sample on other part of the gel was then transferred to a starch gel (a 10% polyacrylamide gel containing 1% soluble starch) using the eBlotTM L1 transfer system (GenScript, Piscataway, NJ, USA). After being renatured by shaking in 20 mM sodium acetate (pH 5.0) containing 0.1% Triton X-100 for 2 h, the starch gel was stained with KI/I2 solution (25 mM KI and 30 mM I2) until reddish area hydrolysis zones appeared.

### 4.10. Analysis of the Enzyme Properties

The optimum pH for the different sources of AmyS was evaluated at 65 °C in 20 mM sodium acetate buffer (pH 2.5 to 12) with 1% soluble starch as substrate for 1 h. The released reducing sugar was measured by the DNS method described above. To determine the optimum temperature of the different sources of AmyS, α-amylase activity was measured at different temperatures in the range of 30–100 °C for 1 h with 1% soluble starch in 20 mM sodium acetate buffer (pH 5.5). The thermostability of α-amylase was assessed by incubating the enzyme at 75 °C. Samples were taken every 10 min for amylase activity assay.

To determine the storage stability of ASC-3 soybean seed at room temperature, amylase activity was measured every 2 months for a period of 1 year. The activity was measured by the DNS method described above. Commercial thermostable α-amylases (N1–N3) severed as controls. N1 (thermostable α-amylase AAS, Sunson, Hebei, China), N2 (Ban480L, Novozymes China, Tianjing, China), and N3 (Termamyl SC DS, Novozymes China, Tianjing, China).

The kinetics of the different sources of AmyS α-amylase proteins were measured at pH 5.5 and 65 °C, varying the starch concentration from 0 to 7.5 mg/mL. All experiments were performed in triplicate, and mean values were used to calculate the Km and Vmax values using the Lineweaver–Burk plotting method.

### 4.11. Analysis Agronomic Traits and Yield Performance of Transgenic Plants

ASC-3 transgenic soybean seeds, along with non-transgenic seeds, were planted in the Experiment Farm of Zhejiang University at Hangzhou, Zhejiang in April 2023 at the T4 generation. At maturity, 40 individual plants of transgenic or non-transgenic soybean were randomly selected for agronomic assessment, including emergence, plant height, number of nodes and branches, pods per plant, total seeds, weight of single or hundred seed, and growth stages. Statistical significance was analyzed using the independent-samples *T* test; values of *p* < 0.05 were considered to be significant.

## 5. Conclusions

In conclusion, the results presented here demonstrate the feasibility, high efficiency, and cost effectiveness of α-amylase AmyS production by transgenic soybean. Moreover, soybean may also serve as a bioreactor for the production of other amylases.

## Figures and Tables

**Table 1:** The enzyme kinetics of three sourced AmyS proteins.

Source	Specific Activity (U/mg)	Kinetic	
		Km (mg/mL)	Vmax (mL/min)
*E. coli* AmyS	17,300	28.71	4.26
Purified soybean AmyS	15,057	25.57	3.70

**Table 2:** Comparison of agronomic traits of the transgenic and non-transgenic soybean under field conditions.

Lines	Title 2	Title 3
Emergence (%)	99.71	99.98
Plant height (cm)	99.75 ± 10.04	103.00 ± 14.03
Nodes	13.19 ± 1.72	15.17 ± 1.72 *
Branches	5.17 ± 0.41	5.16 ± 0.41
Pods of per plant	96.50 ± 39.84	115.17 ± 23.27
Seeds of per plant	203.71 ± 72.66	275.50 ± 69.30
Kernel weight of per plant (g)	53.80 ± 17.91	56.78 ± 24.54
Hundred kernel weight (g)	25.23 ± 0.90	24.20 ± 0.55
Days to maturity (day)	92.0	92.0

Values are given as means (±SD), *n* = 40. Independent-samples *t*-test was used for significance analysis. * represents *p* < 0.05.

## Data Availability

Data is contained within the article or Supplementary Materials.

## Supplementary Materials

The following supporting information can be downloaded at https://www.mdpi.com/article/10.3390/plants13111539/s1: Figure S1: Sequence analysis of soybean expressed AmyS; Figure S2: Lineweaver–Burk enzyme kinetic assay plots for *E. coli* AmyS, soybean AmyS and ASC-3 seed; Figure S3: AmyS transgenic maize seeds and soybean seed; Figure S4: Comparison of α-amylase activity in ASC-3 seeds and commercial thermostable α-amylase products at different temperatures; Table S1: Segregation analysis of AmyS transgenic soybean plants (T_1_).
