# Supplementary material for: Transgenic Soybean for Production of Thermostable α-Amylase
Source: Plants (Basel). 2024 Jun 2;13(11):1539. doi: 10.3390/plants13111539 (PMC11174511; doi:10.3390/plants13111539)
Supplement: Supplementary file 1 [file plants-13-01539-s001.zip › plants-3019667-supplementary.pdf]

Supplementary Material

Table S1. Segregation analysis of AmyS transgenic soybean plants (T_1).

T_0 line	Number of seedlings[a]			χ^2	p	H_0
	**Total**	**Flazasulfuron^R/AmyS$^+$**	**Flazasulfuron^S/AmyS$^-$**	**(3:1)**		***hypothesis*[b]**
ASA-15	24	17	7	0.22	0.64	Accepted
ASA-20	30	24	6	0.4	0.53	Accepted
ASC-3	21	17	4	0.39	0.53	Accepted

[a] The seedlings were detected by AmyS specific PCR. AmyS$^+$ and AmyS$^-$ seedlings were scored.

[b] The observation ratio is considered to fit the expected segregation ratio of 3:1 if P > 0.05.

A	A	P	F	N	G	T	M	M	Q	Y	F	E	W	Y	L	P	D	D	G	T	L	W	T	K	V	A	N	E	A
N	N	L	S	S	L	G	I	T	A	L	W	L	P	P	A	Y	K	G	T	S	R	S	D	V	G	Y	G	V	Y
D	L	Y	D	L	G	E	F	N	Q	K	G	T	V	R	T	K	Y	G	T	K	A	Q	Y	L	Q	A	I	Q	A
A	H	A	A	G	M	Q	V	Y	A	D	V	V	F	D	H	K	G	G	A	D	G	T	E	W	V	D	A	V	E
V	N	P	S	D	R	N	Q	E	I	S	G	T	Y	Q	I	Q	A	W	T	K	F	D	F	N	G	R	G	N	T
Y	S	S	F	K	W	R	W	Y	H	F	D	G	V	D	W	D	E	S	R	K	L	S	R	I	Y	K	F	R	G
I	G	K	A	W	D	W	E	V	D	T	E	N	G	N	Y	D	Y	L	M	Y	A	D	L	D	M	D	H	P	E
V	V	T	E	L	K	N	W	G	K	W	Y	V	N	T	T	N	I	D	G	F	R	L	D	A	V	K	H	I	K
F	S	F	F	P	D	W	L	S	Y	V	R	S	Q	T	G	K	P	L	F	T	V	G	E	Y	W	S	Y	D	I
N	K	L	H	N	Y	I	T	K	T	N	G	T	M	S	L	F	D	A	P	L	H	N	K	F	Y	T	A	S	K
S	G	G	A	F	D	M	S	T	L	M	N	N	T	L	M	K	D	Q	P	T	L	A	V	T	F	V	D	N	H
D	T	E	P	G	Q	A	L	Q	S	W	V	D	P	W	F	K	P	L	A	Y	A	F	I	L	T	R	Q	E	G
Y	P	C	V	F	Y	G	D	Y	Y	G	I	P	Q	Y	N	I	P	S	L	K	S	K	I	D	P	L	L	I	A
R	R	D	Y	A	Y	G	T	Q	H	D	Y	L	D	H	S	D	I	I	G	W	T	R	E	G	V	T	E	K	P
G	S	G	L	A	A	L	I	T	D	G	P	G	G	S	K	W	M	Y	V	G	K	Q	H	A	G	K	V	F	Y
D	L	T	G	N	R	S	D	T	V	T	I	N	S	D	G	W	G	E	F	K	V	N	G	G	S	V	S	V	W
V	P	R	K	T	T	V	S	T	I	A	W	P	I	T	T	R	P	W	T	G	E	F	V	R	W	T	E	P	R
L	V	A	W	P																									
1				5					10					15					20					25					30

Figure S1. Sequence analysis of soybean expressed AmyS. The peptides identified by Nano LC-ESI-MS/MS mass spectrometry are highlighted with red; The N-terminal sequencing using Edman degradation are highlighted with yellow.

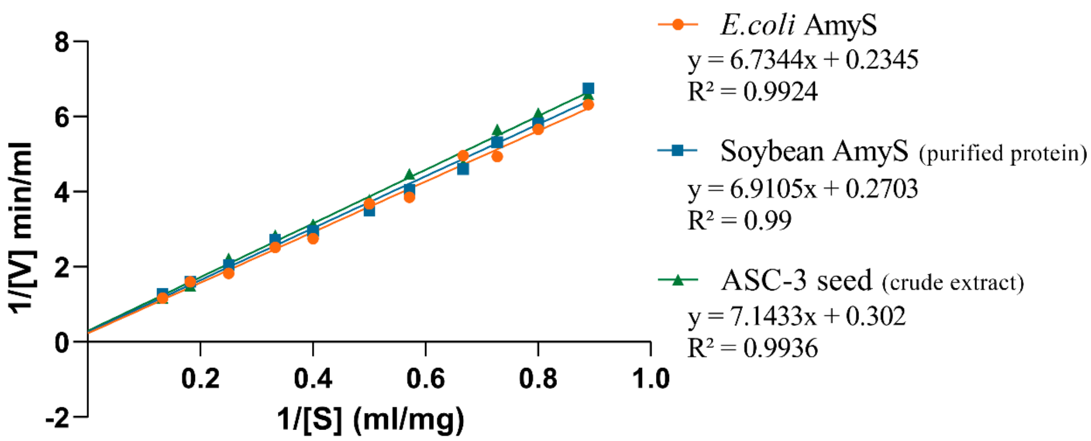

Figure S2. Lineweaver-Burk enzyme kinetic assay plots for *E. coli* AmyS, soybean AmyS and ASC-3 seed. α-amylase activities were assay at pH 5.5 and 65°C using different concentrations of substrate. V represents the initial rate of enzyme reaction and S is substrate concentration.

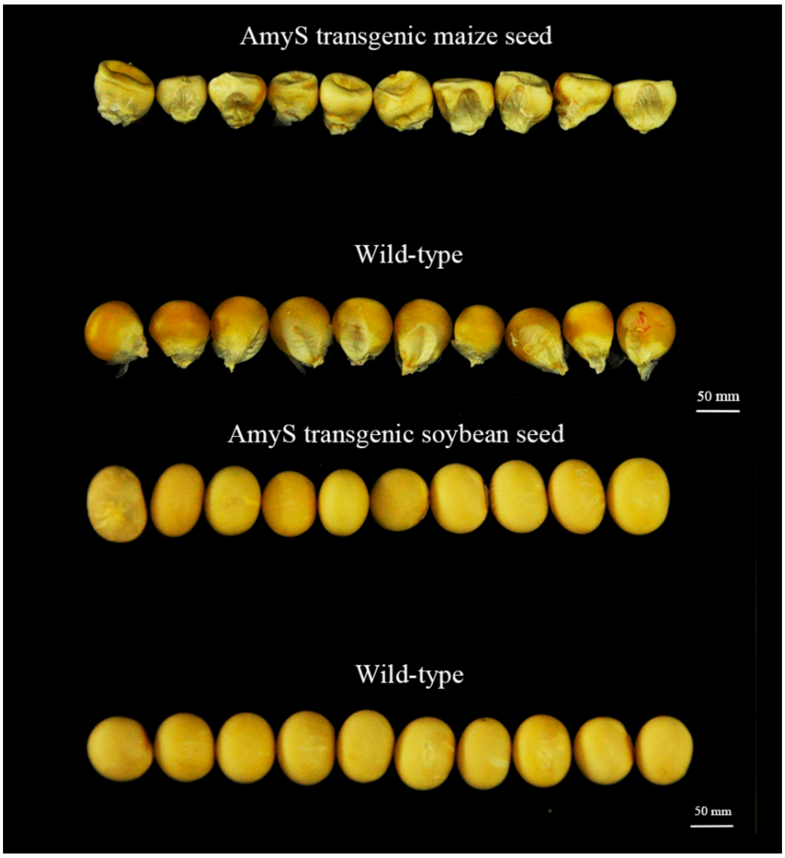

Figure S3. AmyS transgenic maize seeds and soybean seed.

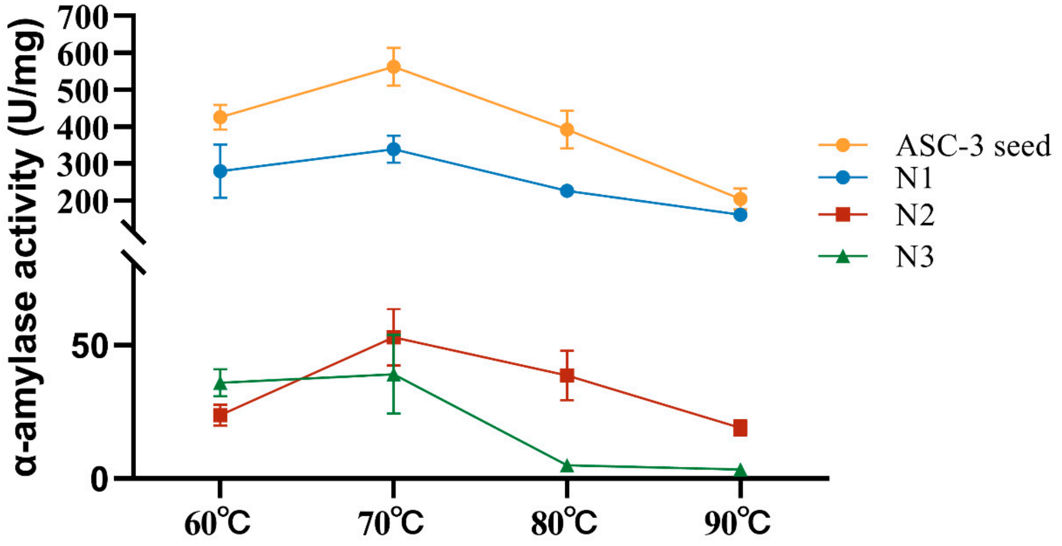

Figure S4. Comparison of α-amylase activity in ASC-3 seeds and commercial thermostable α-amylase products at different temperature. N1 (vendor 1), N2 (vendor 2), N3 (vendor 3).
